# Whole-genome sequencing of *Paenibacillus phoenicis* isolated from the Phoenix Mars Lander spacecraft assembly facility

**DOI:** 10.1128/mra.01265-23

**Published:** 2024-05-14

**Authors:** Andrew Garcia, Romar Rivera, Anna C. Simpson, Nitin K. Singh, Stefan Green, Kasthuri Venkateswaran

**Affiliations:** 1 Jet Propulsion Laboratory, California Institute of Technology, Pasadena, California, USA; 2 Oregon State University, Corvallis, Oregon, USA; 3 Blue Marble Space Institute of Science, Seattle, Washington, USA; 4 Rush University Medical Center, Genomics and Microbiome Core Facility, Chicago, Illinois, USA; Indiana University, Bloomington, Bloomington, Indiana, USA

**Keywords:** cleanroom, whole genome sequencing, planetary protection, endospores, NASA

## Abstract

The genome of *Paenibacillus phoenicis,* a spore-forming bacterium isolated from the spacecraft assembly facility of the Phoenix mission, was generated via hybrid assembly by merging short and long reads. Examining this genome may shed light on strategies to minimize the risk of contaminating extraterrestrial environments with Earth-based microorganisms.

## ANNOUNCEMENT

*Paenibacillus phoenicis* 3PO2SA^T^ (DSM = 27463^T^), an endospore-forming aerobic bacterium, was isolated from the NASA Phoenix Lander spacecraft assembly facility. Its novel taxonomic position has been previously reported (1). The survival capabilities of *P. phoenicis* in nutrient-poor conditions, such as those characterized in spacecraft cleanrooms and the deep subsurface of a molybdenum mine at ~2,141 m depth, are of interest to NASA’s Planetary Protection program due to potential risk that the bacterium could be unintentionally carried on spacecraft, leading to biological contamination of planets that these spacecraft visit. Its recent isolation from blood and cerebrospinal fluid (2, 3) is unusual given previous isolation locations. Due to its potential association with humans, it is possible that this bacterial species could be medically relevant and impact the health of future astronauts.

Strain 3PO2SA^T^ was revived by re-streaking on tryptic soy agar and incubating at 25°C for 3 days. Purified bacterial biomass scraped from agar media using a sterile loop was extracted for DNA using the ZymoBIOMICS DNA MagBead kit per manufacturer’s instructions.

A short-read Illumina library was prepared using an Illumina Nextera XT DNA library prep kit according to the manufacturer’s instructions, and sequenced on an Illumina NovaSeq X instrument with paired-end 2 × 150 base reads. For long-read sequencing, a PacBio SRE XL kit was used to deplete short DNA fragments below 40 kb to produce high-molecular-weight gDNA. A barcoded library was prepared using a Rapid Barcoding Kit 96 from Oxford Nanopore Technologies (ONT) and purified with AMPure XP beads. The barcoded library was loaded onto a MinION R9.4.1 Flow Cell and sequenced on a MinION Mk1B. Base calling was performed using Guppy v.6.5.7. The same DNA sample was used for both platforms.

**Fig 1:**
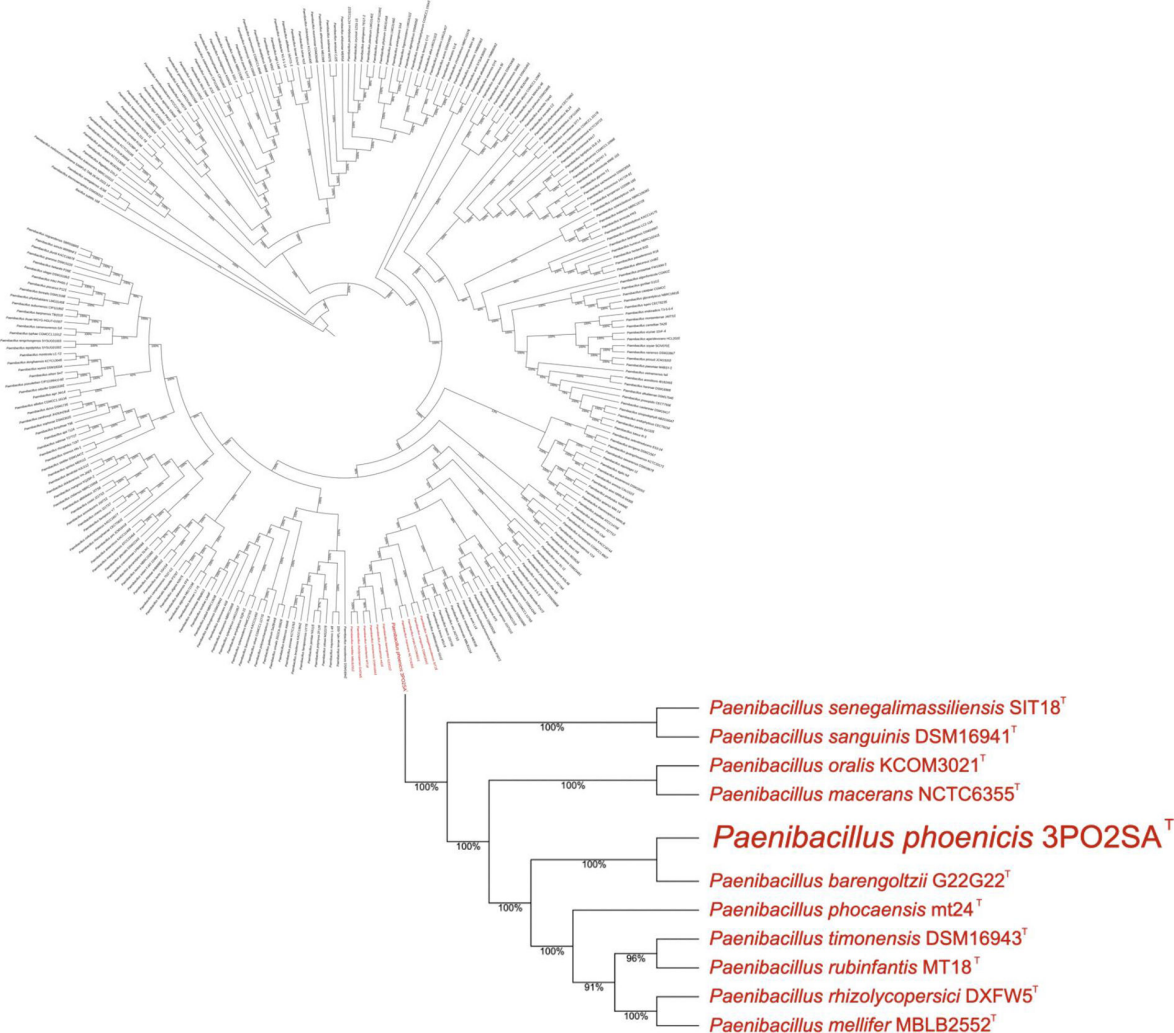
Whole-genome tree of *Paenibacillus phoenicis* with all members of the genus.

A total of 6,396,442 paired Illumina reads and 289,354 ONT reads were generated. N50 for ONT reads was 85,261 bp. All available Illumina reads and 92,000 randomly selected ONT reads were input into assembly. Default parameters were used for all software unless otherwise specified. CLC Genomics Workbench v.23.0.3 (QIAGEN, Aarhus, Denmark) was used to assemble the genome from both short and long reads, using *de novo* long-read assembly followed by long- then short-read polishing, to produce a single complete, closed bacterial chromosome, 4,738,650 bp in length and with 407× coverage. A full-genome tree consisting of all sequenced *Paenibacillus* species (*n* = 268) was made using GToTree v.1.8.3 (4) and was visualized (Fig. 1) with the Interactive Tree of Life v.6.8.1 (5). Assembly statistics were generated via Quast v.5.2.0 (6). The NCBI Prokaryotic Genome Annotation Pipeline (7) was used for annotating protein-coding and functional genes present. The genome contained 4,422 genes where 4,244 were protein-coding. GC content of the genome was 52.69%, with 77 tRNA genes and 22 rRNA genes (seven 5S, eight 16S, and seven 23S copies). The complete genome average nucleotide identity (8) values showed a 91.71% identity match with *P. phoenicis’* closest relative, *Paenibacillus barengoltzii*.

## Data Availability

This Whole Genome Shotgun project PRJNA1055543 has been deposited at DDBJ/ENA/GenBank under the accession JAYERP000000000. The version described in this paper is version 1. The raw reads (SRA) have been deposited under the accession number SRR27319389.
